# Burn-related Collagen Conformational Changes in *ex vivo* Porcine Skin using Raman Spectroscopy

**DOI:** 10.1038/s41598-019-55012-1

**Published:** 2019-12-16

**Authors:** Hanglin Ye, Rahul, Uwe Kruger, Tianmeng Wang, Sufei Shi, Jack Norfleet, Suvranu De

**Affiliations:** 1 Center for Modeling, Simulation and Imaging in Medicine (CeMSIM), Rensselaer Polytechnic Institute, Troy, NY USA; 2 The Department of Chemical and Biological Engineering, Rensselaer Polytechnic Institute, Troy, NY USA; 3 U.S. Army Futures Command, Combat Capabilities Development Command Soldier Center STTC, Orlando, FL USA

**Keywords:** Proteins, Medical research

## Abstract

This study utilizes Raman spectroscopy to analyze the burn-induced collagen conformational changes in *ex vivo* porcine skin tissue. Raman spectra of wavenumbers 500–2000 cm^−1^ were measured for unburnt skin as well as four different burn conditions: (i) 200 °F for 10 s, (ii) 200 °F for the 30 s, (iii) 450 °F for 10 s and (iv) 450 °F for 30 s. The overall spectra reveal that protein and amino acids-related bands have manifested structural changes including the destruction of protein-related functional groups, and transformation from α-helical to disordered structures which are correlated with increasing burn severity. The deconvolution of the amide I region (1580–1720 cm^−1^) and the analysis of the sub-bands reveal a change of the secondary structure of the collagen from the α-like helix dominated to the β-aggregate dominated one. Such conformational changes may explain the softening of mechanical response in burnt tissues reported in the literature.

## Introduction

Thermal injury due to burns induces irreversible changes to the structural components of the skin, causing its loss of functionality to protect the body against external mechanical, physical, chemical and biological factors^1^. Such changes manifest themselves at the continuum level as changes of various material properties, *i.e*. mechanical, thermal, electrical and optical properties *etc*., which can then be utilized to assess burn degrees and monitor wound healing process^2,3^. It is reported that burns cause softening of the skin, which allows discrimination between unburnt and burnt tissues^4^. A detailed understanding of the underlying mechanisms that induce such changes in mechanical properties can improve burn diagnosis and treatments processes.

Collagens are key constituents of mammalian skin, contributing to approximately 75–90% of the dry weight of the dermis layer^5,6^. They provide strength to the skin and determine its mechanical responses in the large deformation regime^6–8^. Hence, investigating the mechanism of burn-induced changes that affect the mechanical properties requires studying associated changes in the collagen structures. It is reported in the literature that collagen fibers undergo denaturation upon heating as they shrink from a native triple helix structure to a more random (coiled) structure, as a result of unfolding and aggregations of protein molecules^9–12^. Thus far, most of the collagen heating studies are limited to the examination of water-soluble collagens, and few studies have examined the structural changes of collagens in burnt skin tissues, especially for those resulted from high temperature burns (>100 °C)^9,13–15^. In this work, we use Raman spectroscopy to examine the conformational changes of collagens in *ex vivo* porcine skin tissue under four different burn conditions (i) 200 °F for 10 s, (ii) 200 °F for 30 s, (iii) 450 °F for 10 s and (iv) 450 °F for 30 s, which are shown to correlate with different burn degrees^4^.

Raman spectroscopy is a non-destructive method for investigation of structural information of materials. It relies on the principle of Raman scattering, where a small fraction (~1 in 10^7^) of the photons are scattered inelastically by an excitation, with the scattered photons undergoing wavelength shifts. The shifts provide a unique characterization of the chemical structure of the specific material, comparable to a molecular fingerprint^16^. Due to its non-invasive nature, it has been increasingly applied for analyzing biological tissues. For skin tissue, Raman spectroscopy has been used to detect skin cancer^17^, investigate the strain-induced protein molecular changes^7^, identify the dermo-epidermal junctions^6^, measure physiological skin parameters of *in vivo* human volar forearm skin including cholesterol, urea, and water^18^, and examine the difference of components such as water, lipids and natural moisturizing factors (NMF) from skin across different body sites^5,19^, *etc*. Recently, Zhai *et al*.^20^ examined the effect between steam burns and dry burns on *ex vivo* porcine ear skin tissues using Raman spectroscopy. Their work, however, focuses mostly on the analysis of the water content in skin which leads to the changes in skin permeability, rather than analyzing the burn-induced collagen structural changes. Rangaraju *et al*.^21^ used Raman spectroscopy in conjunction with OCT to classify burn degrees. Pielesz *et al*.^22^ applied Raman spectroscopy to examine the structural changes of collagen in reaction to L-ascorbic acid (LA) during the burn wound healing process in order to evaluate the therapeutic effect of LA. These works, though related to Raman spectroscopy and skin burn research, did not focus on analyzing burn-induced collagen structural changes, and thus is different from the scope of this study.

The paper is organized as follows. First, we present the methodology aspects of the study, including the sample preparation, experimental protocols and data processing in Section 2. Then, we present our results in Section 3, followed by discussion in Section 4. Conclusions and future work are presented in Section 5.

## Materials and Methods

In this section, we present the methodology aspects of the study. Section 2.1 reports the sample preparation process. The Raman spectroscopy device is briefly described in Section 2.2. The data processing detail is discussed in Section 2.3. The statistical analysis techniques are presented in Section 2.4.

### Sample preparation

Fresh porcine abdominal skin and subcutaneous tissues were locally sourced, stored in sealed plastic bags within a plastic container along with ice cooling pad to ensure freshness, and transported to the lab within the same day of experimentation. The skin was then separated from the underlying muscles using scalpels. Prior to the experiments, the samples were washed with distilled water to ensure minimum contamination from other chemicals.

Biopsy punches with 7 mm diameters were used to harvest the test samples from the skin specimens. The unburnt tissue samples were directly punched out from pieces of skin before burning. For the burnt tissue samples, the pieces of skin were first burnt on a commercial griller (Cuisinart® GR-300WS Griddler Elite Grill, Conair Corporation, NJ)^4^ at the desired temperature for the desired duration, then removed from the heat source and cooled down to room temperature. The burnt tissue samples were then punched out from pieces of burnt skin. All the samples were stored in air-tight glass containers, labeled by their corresponding burn groups, to minimize air exposure, which can cause degradation of the tissue before the Raman testing. For each burn group, we prepared about 20 samples based on literature^21^.

The four burn conditions: (i) 200 °F for 10 s, (ii) 200 °F for 30 s, (iii) 450 °F for 10 s and (iv) 450 °F for 30 s, were chosen based on literature indicating that they may result in second (partial thickness) and third degree (full thickness) burns in *in vivo* porcine tissue^23–25^. Specifically, burning at 200 °F for 10 s can create superficial second degree burns^24^, while burning at 450 °F for 30 s could lead to third degree burns^25^. Burning at 200 °F for 30 s and 450 °F for 10 s are estimated to result in second to third degree burns based on interpolations from plots and experimental data reported in literature^2,4,18,21^. An estimation of energy uptake of the tissues from these burn conditions is provided based on the analytical solution of 1D heat transfer equation (Supplementary Information). Calculation shows that the energy uptake of the tissues increases monotonically from group (i) to group (iv). In addition, group (ii) 200 °F for 30 s, and (iii) 450 °F for 10 s have similar amount of heat generation in comparison with other groups.

### Raman spectroscopy

Raman spectra were measured with a Renishaw confocal Raman spectroscope (Renishaw plc. UK). A 785 nm laser (diode type) with an output of 200 mW was used as the excitation source. The wavenumber was calibrated with silicone before each test. Raman spectra were collected at multiple locations for each sample with an integration time of 10 s and 3 accumulations. Spectra with high cosmic noise were discarded before analysis. In the end, we obtained in total 102 spectra (*N*_*unburnt*_ = 23, *N*_200*F*10*s*_ = 18, *N*_200*F*30*s*_ = 20, *N*_450*F*10*s*_ = 20, *N*_45*F*30*s*_ = 21) for analysis. Spectra were then processed with background subtraction, normalization, and deconvolution, which will be described in the following sections.

### Data processing

#### Background subtraction and normalization

The Raman spectrum of skin usually contains background signals due to skin fluorescence^16^. Though near-infrared excitation (785 nm) is applied in order to minimize the background, it is still necessary to remove the background through signal processing, since the background signal will largely affect the subsequent classification accuracy^17^. To subtract the background, a polynomial fit with an asymmetric truncated quadratic function as the cost function was performed for all the spectra^26^.

After background subtraction, normalization was performed to bring all the spectra to the same interpretable scale. Vector normalization is one of the most common methods^27^, where all the spectra are normalized with respect to their magnitude. Specifically, if the Raman shift is stored in a vector, ***s*** = [*s*_1_, *s*_2_, *s*_3_, …, *s*_*N*_] for a single spectrum, where *N* is the number of data points, vector normalization is performed as

1 $${{\boldsymbol{s}}}_{nor}={\boldsymbol{s}}/{\Vert {\boldsymbol{s}}\Vert }_{2}$$

where $${\Vert {\boldsymbol{s}}\Vert }_{2}=\sqrt{{s}_{1}^{2}+{s}_{2}^{2}+\ldots +{s}_{N}^{2}}$$ ^28^.

#### Deconvolution

Deconvolution was performed with the commercial software Origin (OriginLab). This involves decomposition of a band into multiple sub-bands in order to analyze the contribution from each component^12^. The target in this study is the amide I region (1580–1720 cm^−1^) as it is commonly used for analysis of protein secondary structure^1^. The sub-band positions and numbers were chosen based on reported values in the literature^1,7^. Here, we used 10 sub-bands for the fitting with initial positions of 1605, 1616, 1625, 1633, 1644, 1653, 1662, 1677, 1685, 1693 cm^−1^. The shape of each band was approximated by a Lorentz function. The goodness of the fit is evaluated with adjusted *R*^2^ values, which is calculated as,

2 $${R}_{adj}^{2}=1-[\frac{(1-{R}^{2})(n-1)}{(n-k-1)}]$$

where *n* is the number of points in the data sample and *k* is the number of independent regressors, *i.e*. the number of variables in the model, excluding the constant. ${R}^{2}\equiv 1-\frac{S{S}_{res}}{S{S}_{tot}}$ is the *R*^2^ values, with *SS*_*res*_ being the regression sum of squares and *SStot* being the total sum of squares of the data.

## Results

### Raman spectra for all the burn groups

**Figure 1:**
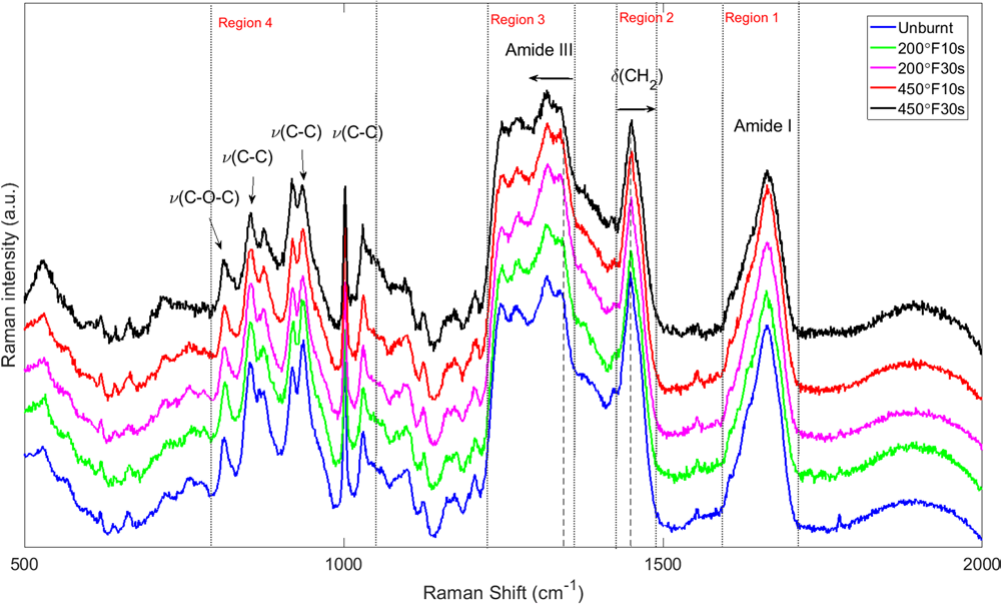
Mean Raman spectra of *ex vivo* porcine skin (unburnt and burnt with different burnt severities). Major band assignments are indicated. Abbreviations: *ν*, stretching mode; *δ*, in-plane bending mode.

The mean Raman spectra of 5 groups (unburnt + 4 burn groups) are presented in Fig. 1. Major bands and their assignments are also indicated. Detailed band assignments are listed and explained in Table 1. From Fig. 1, we can broadly identify four major regions (wavenumber from high to low): ~1590–1710, ~1420–1490, ~1220–1350, and ~800–1030 cm^−1^. These regions contain major characteristic bands in the skin, dominated by different vibrational spectra. Region 1 is dominated by the amide I band, contributed mainly by the vibrations of C=O stretching^7,12^. It is associated with the amide vibrations of the polypeptide chains, which lead to the formation of proteins (collagens). The amide I band is most commonly used for the analysis of secondary structure of proteins to which they are highly sensitive^12^. Region 2 is dominated by the vibrations of CH_2_ bending, which are associated with components such as proteins and lipids. Regions 3 is dominated by the amide III bands, which are also characteristic bands associated with polypeptide chains, and are mainly associated with vibrations of C–N stretching and N–H bending^7,12^. Unlike the amide I band, amide III bands are not suitable for analysis of secondary structures they are affected by side-chain structures, and thus are overlapped by other bands^1,12^. Region 4 is dominated by *ν*(C–C) vibrations and are mainly assigned to amino acids such as proline, hydroxyproline, tyrosine and tryptophan^29^. It can be seen that spectra of the skin are dominated by vibrational spectra from proteins, lipids and amino acids^7^. In order to study the burn-induced molecular structures of collagens, we focus our attention on amino acid and protein related bands.

**Table 1:** Major band assignments in *ex vivo* porcine skin Raman spectra.

Band positions (cm^−1^)	Assignments	Structure	Reference values (cm^−1^)
813	*ν*(C–C) *ν*(C–O–C)	Protein backbone, collagen cross-link, proline, hydroxyproline, tyrosine	813–817^6,7,29^
853	*ν*(C–C)	Proline, hydroxyproline, tyrosine	853–856^6,7,29^
873	*ν*(C–C)	Hydroxyproline, tryptophan, protein	873–879^6,7,29^
919	*ν*(C–C)	Proline	919–920^6,7,29^
937	*ν*(C–C)	Protein (collagen), α-helix, proline, hydroxyproline	933–938^6,7,29^
1002	*ν*(C–C)	Phenylalanine (collagen), protein	1000–1005^6,7,29^
1030	*ν*(C–C), δ(C–H), *ν*(C–N), δ(CH_2_CH_3_)	Phenylalanine (collagen), lipids, proteins	1030–1034^6,29^
1247	*ν*(C–N), δ(N–H)	Amide III, proteins	1242–1255^7,29^
1270	*ν*(C–N), δ(N–H)	Amide III (collagen), α-helix	1270–1280^7,29,38^
1317	δ(CH_3_CH_2_)	Collagen, nuclei acids	1322^29^
1342	δ(C–H), δ(CH_3_CH_2_)	Collagen, protein, lipids	1330–1340^7,29^
1448	δ(CH_2_), δ(CH_2_CH_3_)	Collagen, protein, lipids	1440–1450^7,29^
1662	*ν*(C=O), *ν*(C=C), *ν*(C–N), δ(N–H)	Amide I (collagen)	1650–1676^7,29^

Abbreviations: ν, stretching mode; δ, in-plane bending mode.

### Burn-induced spectral changes

**Figure 2:**
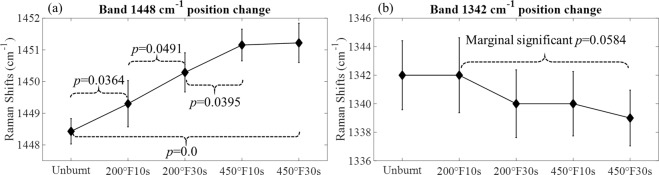
Burn-induced band position changes. (**a**) Band 1448 cm^−1^ (**b**) Band 1342 cm^−1^. Values are presented as mean ± 95% confidence interval. *p* values are reported for those pairs of groups that show statistical significance.

**Figure 3:**
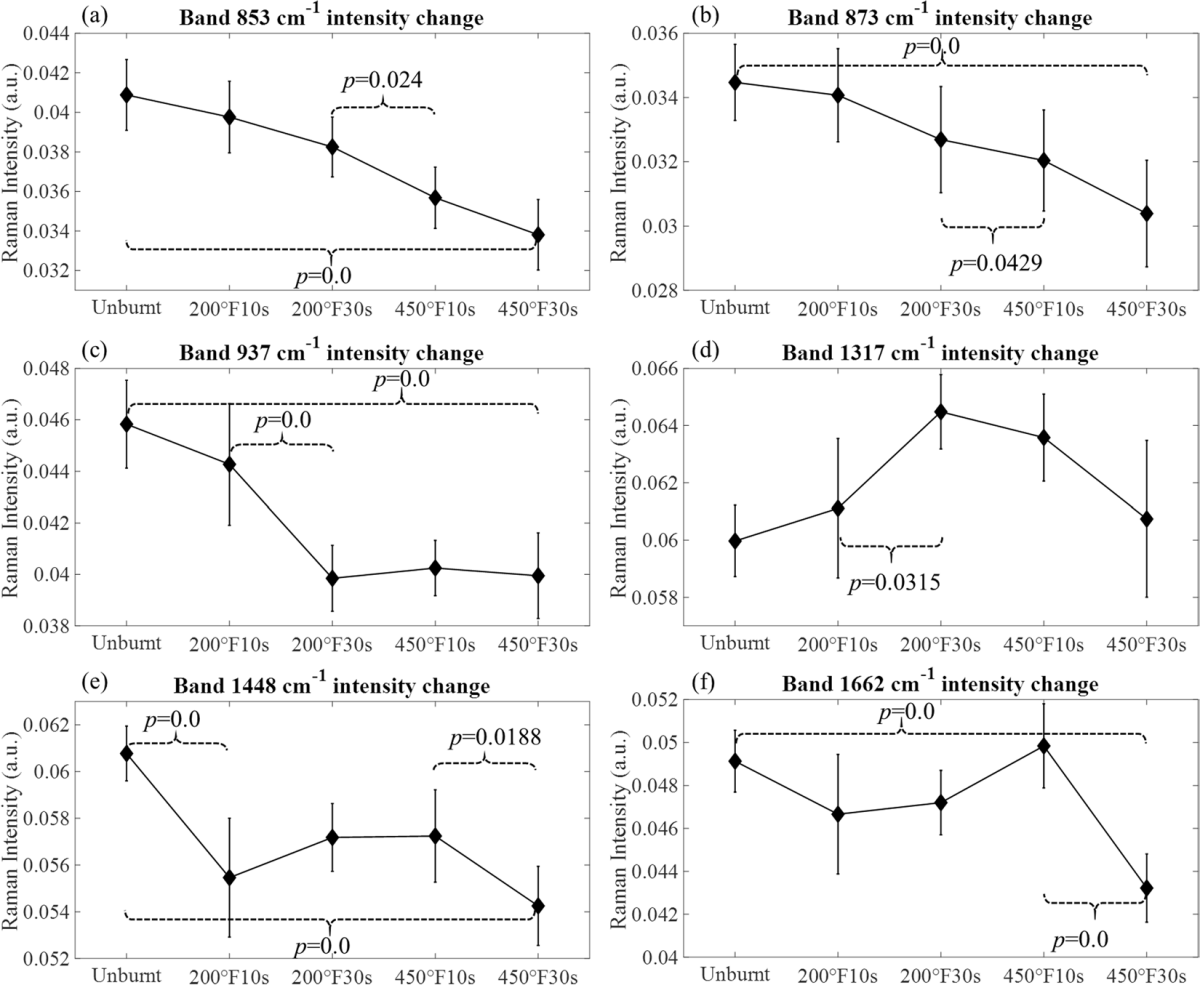
Burn-induced band intensity changes. (**a**) Band 853 cm^−1^, (**b**) Band 873 cm^−1^, (**c**) Band 937 cm^−1^, (**d**) Band 1317 cm^−1^, (**e**) Band 1448 cm^−1^, (**f**) Band 1662 cm^−1^. Values are presented as mean ± 95% confidence interval. *p* values are reported for those pairs of groups that show statistical significance.

Both position and intensity changes are observed in certain bands of the spectra. As shown in Fig. 1, position shifts happen in bands 1448 and 1342 cm^−1^ in the amide III region. Such changes of these two bands are plotted in Fig. 2. The intensity changes are observed in bands 853, 873, 937, 1317, 1448, and 1662 cm^−1^, and are plotted in Fig. 3. For each neighboring pair of burn groups (*i.e*. Unburnt vs. 200 °F10s, 200 °F10s vs. 200 °F30s, …, *etc*.), as well as the Unburnt vs. 450 °F30s group, two sample t tests are performed to evaluate the statistical significance of the change (H_0_: *μ*_A_ = *μ*_*B*_, H_a_: *μ*_A_ ≠ *μ*_*B*_, where *μ* indicates the mean value of data, and *A*, *B* are two different burn group). *p* values are reported for those that are statistical significant.

From Fig. 2 we can see that with increasing burn severity, there is a gradual shift towards higher wavenumbers for the 1448 cm^−1^ band, and a shift towards lower wavenumbers for the 1342 cm^−1^ band. Band 1448 cm^−1^ attributes mainly to the assignment of CH_2_ bending associated with lipids and proteins, while band 1342 cm^−1^ attributes mainly to the assignment of C-H bending within the amide III bands, which are associated not only with proteins, but also other possible side chains. Their shift is consistent with results reported in this prior work (Olsztyńska-Janus *et al*.)^1^, which can be an indication of structural changes in lipids and proteins. The position changes in band 1448 cm^−1^ is confirmed by the two sample t tests, which indicates that there is a significant change between the two end groups (Unburnt vs. 450 °F30s), and three out of four changes of the in-between groups is also significant. The comparison between the last two groups (450 °F10s vs. 450 °F30s) does not show significant changes in band 1448 cm^−1^ position, which may indicate possible plateauing of the change. For the band 1342 cm^−1^, though there is no statistically significant changes between the two end groups, a marginally significant *p* value (*p* = 0.0584) exists for the 200 °F10s vs. 450 °F30s groups. Considering the results reported in the prior work (Olsztyńska-Janus *et al*.)^1^, such change in amide III region is worth noticing and worth examining with further increasing burn severities.

From Fig. 3, we can see that three of the bands (band 853, 873 and 937 cm^−1^) present a decreasing trend in intensities. Specifically, the intensities of band 853 and 873 cm^−1^ decrease monotonously with increasing burn severities, which is also confirmed by the statistically significant changes between the two end groups (Unburnt vs. 450 °F30s) and in-between groups (200 °F30s vs. 450 °F10s). They correspond to the C–C stretching associated with amino acids, indicating possible changes in the amount of proline/hydroxyproline residues. Band 937 cm^−1^ shows a decreasing trend of intensity in the first three groups, and then stays constant with minor fluctuation, as the burn severity increases, which is again confirmed by the statistically significant changes between the two end groups (Unburnt vs. 450 °F30s) and in-between groups (200 °F10s vs. 450 °F30s), as well as the non-significant *p* values of the last three groups. This is associated with the C–C stretching of the backbone formed by Gly-X-Y sequence^30^. The changes in bands 1317, 1448 and 1662 cm^−1^ are nonmonotonic. Band 1317 cm^−1^ first shows a significant increase in intensity for the 200 °F10s vs. 200 °F30s groups, then decreases back to nearly the original values, resulting in a non-significant change between the two end groups. Since it is associated with the amide III mode, this change may indicate the combined effect of multiple structural changes from the side chains. Band 1448 cm^−1^ shows significant decreases for Unburnt vs. 200 °F10s, and 450 °F10s vs. 450 °F30s as well as the two end groups, with a slight increase in between, which can be related to the structural changes in lipids^1^. Band 1662 cm^−1^ shows significant decrease for 450 °F10s vs. 450 °F30s group and the two end groups, with an increase in the between groups. It corresponds to the amid I mode and may indicate a combined effect from changes of protein secondary structure. In the next section, we will examine the amide I band in detail using deconvolution. It is worth mentioning that, based on the energy uptake estimation provided in Supplementary Information, 200 °F30s and 450 °F10s produce similar amount of heat, which may result in similar level of structural changes of skin components, reflected as the similar change in position of band 1342 cm^−1^, and in intensity of band 937 and 1448 cm^−1^.

### Deconvolution of the amide I band

As is mentioned in the previous section, the amide I band (~1662 cm^−1^) is most commonly used for analysis of protein secondary structures. The secondary structures of skin collagen are mainly α-like helices, β-sheets, β-turns and random coils (disordered)^31,32^. They are closely related to the formation of collagens (type I and type IV) in the skin. Here, we analyze the secondary structure of skin collagens by deconvolution of the amide I band.

**Figure 4:**
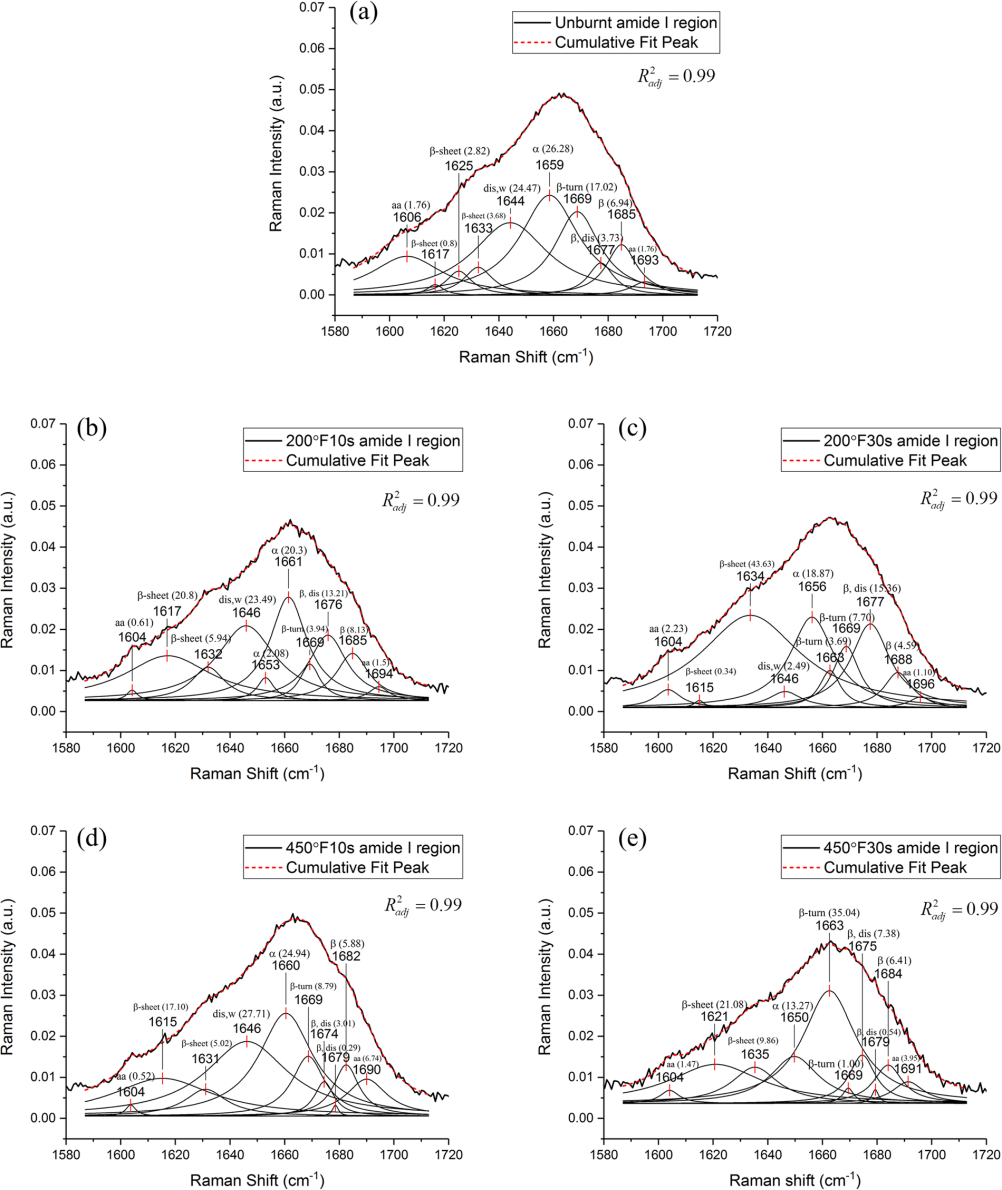
Deconvolution of the amide I region of the Raman spectra of *ex vivo* porcine skin. (**a**) Unburnt skin, (**b**) Skin burnt at 200 °F for 10s, (**c**) Skin burnt at 200 °F for 30 s, (**d**) Skin burnt at 450 °F for 10 s, (**e**) Skin burnt at 450 °F for 30 s. The percentage of area under each curve is shown in bracket. Abbreviations: α – α-like helix, β – β sheets and β turns, dis – disordered structures, w – free and hydrogen bonded water, aa – amino acids.* R*^2^_*adj*_ = 0.99 for all the cases, indicating good fit, and the fitting curves are presented along with the data curve for visualization of the fitted results.

The sub-band number and locations are chosen based on those reported in the literature^1,7^. Table 2 lists the sub-bands in unburnt tissue obtained from this work and those reported in the literature. The deconvolution results are shown in Fig. 4. The sub-band positions and assignments of the unburnt tissue are consistent with data from the literature. The adjusted *R*^2^ value are 0.99 for all the deconvolutions, indicating good fit.

**Table 2:** Sub-band positions and assignments for the amide I region for the unburnt skin tissue.

Sub-band positions for amide I (cm^−1^)	Secondary structure	Reference values
1617	β-sheets	1618^1^
1625	β-sheets	1624, 1628^1,7^
1633	β-sheets	1630^1^
1644	Water, disordered structure	1640–1645^1,7^
1659	α-like helix	1657–1660^32^
1669	β-turns	1664, 1669^1^
1677	β-sheets and turns, disordered structure	1676^7^
1685	β-sheets and turns,	1680, 1686^7,31,39^

The deconvolutions of spectra for burnt tissue show changes in sub-band positions, intensities and areas under curves (Fig. 4(b–e)). Some of these changes can be correlated with the burn severities. Here we focus on the α-like helix band (1659 cm^−1^), the β-turns band (1669 cm^−1^), the water and disordered structure band (1644 cm^−1^), and the β-sheets band (1633 cm^−1^). The α-like helix band (1659 cm^−1^) splits into two bands (1653 and 1661 cm^−1^) in the 200 °F10s group, and the overall area under the curves decreases compared to the unburnt stage. In the 200 °F30s group, its position shifts to 1656 cm^−1^ and its area under the curve also decreases. In the 450 °F10s group, when compared with the unburnt stage, the position of the α-helix band does not change much, while its area under the curve decreases. In the 450 °F30s group, the α-helix band shifts largely towards the lower wavenumber (1650 cm^−1^), accompanied by a decreased area under the curve. The β-turns band (1669 cm^−1^) does not change positions for all the burn groups, and a new band (1663 cm^−1^) appears in groups 200 °F30s and 450 °F30s. The water band (1644 cm^−1^) changes its position to 1646 cm^−1^ in group 200 °F10s, 200 °F30s, and 450 °F10s, and disappears in group 450 °F30s. Its area under the curve in general decreases, with a slight increase in group 450 °F10s. The β-sheets band (1633 cm^−1^) has varying band positions for different burn groups. Its area under the curve remains mostly the same for groups 200 °F10s and 450 °F10s while increasing for groups 200 °F30s and 450 °F30s. These changes will be discussed in detail in the discussion section.

## Discussion

### Burn-induced structural component changes in *ex vivo* porcine skin tissue

As shown in Sections 3.1 and 3.2, the Raman spectra of skin are dominated by vibrational spectra of proteins, lipids, and amino acids, and burn-induced structural changes of these components are reflected as changes in positions and intensities of certain bands. A gradual shift towards higher wavenumber is observed for band 1448 cm^−1^, which is consistent with the literature^1^. A decrease in intensity is also observed for this band. Since this band is associated with CH_2_ bending mode for lipids and proteins, the decrease of intensity can be interpreted as a lowering of the concentration of lipids and/or proteins^33^, resulting from the reduction of CH_2_ groups due to burning. A gradual shift towards lower wavenumber is observed for band 1342 cm^−1^, which is associated with the C–H bending mode. This shift can be viewed as a change of secondary structures from α-helices to disordered structures, as the α-helix is represented by 1300–1340 cm^−1^ whilst the disordered structures are represented by 1260 cm^−1^ in amide III bands^31^. Hence, an increased thermal injury may be related to the destruction of certain lipid/protein related groups, and the transformation of the secondary structures of proteins to disordered ones.

The decreasing intensities of 853, 873 and 937 cm^−1^ indicates a decrease in the proline/hydroxyproline residues. This can be an indication of a reduction in polypeptide chains in tropocollagen as individual polypeptide chains possess an (X-Y-Gly)_n_ pattern, where X and Y are frequently occupied by proline and hydroxyproline, respectively^34^. These amino acids also play important roles in forming stabilizing interactions between tropocollagens by establishing networks of hydrogen bonds with water^35^. Hence they allow tropocollagens to present stable triple-helical conformations. Tropocollagens are further associated with each other by crosslinks to form collagens (type I and type IV in skin)^6^, thus the instability of tropocollagens resulting from reduction of such amino acids can lead to the instability of collagens. The hydroxyproline also increases the thermal stability of collagens^36^, rendering them more resistant to unfolding upon heating^37^. Burning causes a reduction of proline/hydroxyproline in the skin, which can lead to loose triple-helical structures of tropocollagens and less thermally stable collagens. It is worth noting that unlike band 853 and 873 cm^−1^ who present a monotonic decreasing trend, band 937 cm^−1^ shows a significant decrease in the 200 °F30s group, and stays plateau as the burn severity increases. While the similar responses in the 200 °F30s group and the 450 °F10s group can be understood as a result of similar energy uptake of the tissue (see Supplementary Information for details), the “450 °F10s to 450 °F30s” plateau indicates an inert response to further increasing burn severities. One possible explanation for this may be that some of the collagen has undergone denaturation and become gelatin, which is an end product of thermally denatured collagens^13^. Thus, the structural changes reflected by band 937 cm^−1^ which is associated with the C-C stretch mode of the X-Y-Gly backbone^6^ become stable and less prone to abrupt changes due to the fact that collagen has been denatured^13^. However, this conjecture requires further experimentation, which is left as future work.

To summarize, the changes of positions and intensities of certain bands in the skin Raman spectra reveal that burning leads to more disordered, less stable conformations of collagens, as a result of the destruction of protein-related functional groups and amino acids.

### Burn-induced secondary structure changes in skin collagens

The deconvolution of amide I region of the spectra provides insight into the changes of collagen secondary structures. In the unburnt tissue, the amide I band is dominated by the α-like helix, which takes up 26.28% of the total area. The α-helical structures provide strength to the collagen. With an increase in burn severity, the α-like helix band shifts towards lower wavenumbers, indicating denaturation of collagens^1,31^. The reduction in areas under the curves indicates a decreased amount of α-like helices. Instead, an increase in the number of β-turns and sheets (1663 and 1669, 1633 cm^−1^) is observed in burn groups. In the most severe burn group (450 °F30s), the β-turns (1663 cm^−1^) become the dominating structures. The reduction of α-like helices and the increase of β-turns and sheets demonstrate the unfolding of polypeptide chains and the formation of β-aggregates upon heating, which usually accompanies the denaturation process^1,12^. In general, the β structures provide flexibilities to the collagen molecules, which may explain the softening responses observed in burnt skin tissues^4^. The shifting and reduction of water band dominated by the –OH bending (1644 cm^−1^) are an indication of a weakening of hydrogen bonds^1^. The hydrogen bonds between water and hydroxyprolines are essential to the stabilizing interaction that ensures the triple-helical conformation of tropocollagens^6^, a reduction of which can lead to less thermally stability of collagens. By analyzing the changes in collagen secondary structures, we can see that burning leads to more disordered and less stable structures of collagens.

## Conclusion

In this work, we study the burn-induced conformational changes of collagens using Raman spectroscopy. Controlled burning experiments on fresh *ex vivo* porcine skin tissue were performed under four different burning conditions, and the Raman spectra from all the burn groups as well as the unburnt condition were collected. Analysis of the changes in positions and intensities of the protein and amino acids-related bands reveals that collagens in skin transform to more disordered and less thermally stable structures upon burning, that may be less resistant to unfolding. Deconvolution of the amide I region and the corresponding analysis of collagen secondary structures show transitions of α-helices to the β-aggregates and weakening of hydrogen bonds due to thermal injury, which lead to more disordered and less stable structures of collagens in the skin. Such conformational changes may explain the softening responses observed in burnt skin tissues reported in^4^. As future work, *in vivo* studies are planned in order to capture the more complex dynamic changes that occur to the cellular and parenchymal elements of skin post-burn.

## Supplementary information

Supplementary information

## Data Availability

The datasets generated during and/or analyzed during the current study are available from the corresponding author on reasonable request.

## References

[1] Olsztyńska-Janus S, Pietruszka A, Kiełbowicz Z, Czarnecki MA (2018). ATR-IR study of skin components: Lipids, proteins and water. Part I: Temperature effect. Spectrochim. Acta - Part A Mol. Biomol. Spectrosc..

[2] Ye H, De S (2017). Thermal injury of skin and subcutaneous tissues: A review of experimental approaches and numerical models. Burns.

[3] Clark JA, Cheng JCY, Leung KS (1996). Mechanical properties of normal skin and hypertrophic scars. Burns.

[4] Ye H, Rahul, Dargar S, Kruger U, De S (2018). Ultrasound elastography reliably identifies altered mechanical properties of burned soft tissues. Burns.

[5] Caspers PJ, Lucassen GW, Wolthuis R, Bruining HA, Puppels GJ (1998). *In vitro* and *in vivo* Raman spectroscopy of human skin. Biospectroscopy.

[6] Nguyen TT (2013). Characterization of type I and IV collagens by Raman microspectroscopy: Identification of spectral markers of the dermo-epidermal junction. Adv. Biomed. Spectrosc..

[7] Gasior-Głogowska M (2013). FT-Raman spectroscopic study of human skin subjected to uniaxial stress. J. Mech. Behav. Biomed. Mater..

[8] Holzapfel, G. A. Biomechanics of soft tissue. In *The Handbook of Materials Behavior Models* 1049–1063 (Academic, 2001).

[9] Wright NT, Humphrey JD (2002). Denaturation of collagen via heating: An irreversible rate process. Annu. Rev. Biomed. Eng..

[10] Lee JM, Pereira CA, Abdulla D, Naimark WA, Crawford I (1995). A multi-sample denaturation temperature tester for collagenous biomaterials. Med. Eng. Phys..

[11] Theodossiou T (2002). Thermally induced irreversible conformational changes in collagen probed by optical second harmonic generation and laser-induced fluorescence. Lasers Med Sci.

[12] Barth A, Zscherp C (2002). What vibrations tell us about proteins. Q. Rev. Biophys..

[13] Frushour BG, Koenig JL (1975). Raman scattering of collagen, gelatin, and elastin. Biopolymers.

[14] Bryan MA (2007). FTIR studies of collagen model peptides: Complementary experimental and simulation approaches to conformation and unfolding. J. Am. Chem. Soc..

[15] Tiktopulo EI, Kajava AV (1998). Denaturation of Type I collagen fibrils is an endothermic process accompanied by a noticeable change in the partial heat capacity. Biochemistry.

[16] Franzen L, Windbergs M (2015). Applications of Raman spectroscopy in skin research - From skin physiology and diagnosis up to risk assessment and dermal drug delivery. Adv. Drug Deliv. Rev..

[17] Sigurdsson S (2004). Detection of skin cancer by classification of Raman spectra. IEEE Trans. Biomed. Eng..

[18] Binder L (2017). Confocal Raman spectroscopy: *In vivo* measurement of physiological skin parameters – A pilot study. J. Dermatol. Sci..

[19] Caspers PJ, Lucassen GW, Puppels GJGJ (2003). Combined *in vivo* confocal Raman spectroscopy and confocal microscopy of human skin. Biophys. J..

[20] Zhai L (2018). Prediction of steam burns severity using Raman spectroscopy on *ex vivo* porcine skin. Sci. Rep..

[21] Rangaraju Lakshmi Priya, Kunapuli Gautam, Every Dayna, Ayala Oscar D., Ganapathy Priya, Mahadevan-Jansen Anita (2019). Classification of burn injury using Raman spectroscopy and optical coherence tomography: An ex-vivo study on porcine skin. Burns.

[22] Pielesz A (2017). The role of topically applied L-ascorbic acid in *ex-vivo* examination of burn-injured human skin. Spectrochim. Acta - Part A Mol. Biomol. Spectrosc..

[23] Abraham JP, Plourde B, Vallez L, Stark J, Diller KR (2015). Estimating the time and temperature relationship for causation of deep-partial thickness skin burns. Burns.

[24] Cuttle L (2006). A porcine deep dermal partial thickness burn model with hypertrophic scarring. Burns.

[25] Branski LK (2008). A porcine model of full-thickness burn, excision and skin autografting. Burns.

[26] Mazet V, Carteret C, Brie D, Idier J, Humbert B (2005). Background removal from spectra by designing and minimising a non-quadratic cost function. Chemom. Intell. Lab. Syst..

[27] Butler HJ (2016). Using Raman spectroscopy to characterize biological materials. Nat. Protoc..

[28] Gautam R, Vanga S, Ariese F, Umapathy S (2015). Review of multidimensional data processing approaches for Raman and infrared spectroscopy. EPJ Tech. Instrum..

[29] Movasaghi Z, Rehman S, Rehman IU (2007). Raman spectroscopy of biological tissues. Appl. Spectrosc. Rev..

[30] Zhang Q (2011). Raman microspectroscopic and dynamic vapor sorption characterization of hydration in collagen and dermal tissue. Biopolymers.

[31] Wen Z-Q (2007). Raman spectroscopy of protein pharmaceuticals. J. Pharm. Sci..

[32] Belbachir K, Noreen R, Gouspillou G, Petibois C (2009). Collagen types analysis and differentiation by FTIR spectroscopy. Anal. Bioanal. Chem..

[33] Chan JW (2006). Micro-Raman spectroscopy detects individual neoplastic and normal hematopoietic cells. Biophys. J..

[34] Xiao Y (2008). Wavelength-dependent conformational changes in collagen after mid-infrared laser ablation of cornea. Biophys. J..

[35] Shoulders MD, Raines RT (2010). Collagen structure and stability. Annu. Rev. Biochem..

[36] Berg RA, Prockop DJ (1973). The thermal transition of a non-hydroxylated form of collagen. Evidence for a role for hydroxyproline in stabilizing the triple-helix of collagen. Biochenical Biophys. Res. Commun..

[37] Rochdi A, Foucat L, Renou JP (1999). Effect of thermal denaturation on water-collagen interactions: NMR relaxation and differential scanning calorimetry analysis. Biopolymers.

[38] Barry BW, Edwards HGMM, Williams AC, Williams. AC (1992). Fourier transform Raman and infrared vibrational study of human skin: assignment of spectral bands. J. Raman Spectrosc..

[39] King DR (2015). Surgical wound debridement sequentially characterized in a porcine burn model with multispectral imaging. Burns.

