# Supplementary material for: Burn-related Collagen Conformational Changes in ex vivo Porcine Skin using Raman Spectroscopy
Source: Sci Rep. 2019 Dec 16;9:19138. doi: 10.1038/s41598-019-55012-1 (PMC6915721; doi:10.1038/s41598-019-55012-1)
Supplement: Supplementary file 1 — Supplementary information [file 41598_2019_55012_MOESM1_ESM.pdf]

Burn-related Collagen Conformational Changes in *ex vivo* Porcine Skin using Raman Spectroscopy

Hanglin Ye[a], Rahul[a], Uwe Kruger[a], Tianmeng Wang[b], Sufei Shi[b], Jack Norfleet[c] and Suvranu De[a,*]
[a]Center for Modeling, Simulation and Imaging in Medicine (CeMSIM),
Rensselaer Polytechnic Institute, Troy, NY, USA
[b]The Department of Chemical and Biological Engineering,
Rensselaer Polytechnic Institute, Troy, NY, USA
[c]U.S. Army Futures Command, Combat Capabilities Development Command Soldier Center STTC,
Orlando FL, USA

[*] Corresponding author, Tel: +1 (518) 276-6351; Fax: +1 (518) 276-6025; Email: des@rpi.edu

Supplementary Information:

In this Supplementary Information, we provide an analytical solution of a 1D heat transfer equation to estimate the energy uptake of the tissue corresponding to different burn conditions. Though a 1D heat transfer problem is an idealization of the actual burning scenarios, it provides insights into different burn conditions associated with different time and temperate of burn.

The heat flux (q) in the tissue is related to the temperature field $T(x,\mathrm{t})$ based on Fourier's law:

$$q = -k\frac{\partial T(x,\mathrm{t})}{\partial x} \tag{S.1}$$

where k is the thermal conductivity. The temperature field $T(x,\mathrm{t})$ is obtained by solving the 1D conduction equation,

$$\frac{\partial^2 T}{\partial x^2} = \frac{1}{\alpha_h}\frac{\partial T}{\partial t} \tag{S.2}$$

subject to

$$T(0,t) = T_A \tag{S.3}$$

$$\partial T(L,t)/\partial x = -h\left[T(L,t) - T_r\right] \tag{S.4}$$

and

$$T(x,0) = T_r \tag{S.5}$$

where $\alpha_h = k/(C_p\rho)$ is the thermal diffusivity, with C_p being the specific heat, k the thermal conductivity and ρ the density. h is the convection heat transfer coefficient. Equation (S.3) and (S.4) are the boundary conditions of the problem, stating that one end of the sample ($x = 0$) is subjected to the desired applied temperature T_A while the other end ($x = L$) is radiating heat into a surrounding with room temperature T_r. Equation (S.5) states that the sample is at room temperature at time $t = 0$.
The solution to this system is given as

$$T(x,\mathrm{t}) = T_A + \frac{h(T_r - T_A)}{1+hL}x + \sum_{n=1}^{\infty} B_n e^{-(\lambda_n \alpha_h t)} \sin(\sqrt{\lambda_n}x)\text{ , } n=1,2,3,.. \tag{S.6}$$

with

$$B_n = \frac{\int_0^L \left(T_r - \left(\frac{h(T_r - T_A)}{1+hL}x + T_A\right)\right)\sin(\sqrt{\lambda_n}x)dx}{\int_0^L \sin^2\left(x\sqrt{\lambda_n}\right)dx} = \frac{-\frac{(\mathrm{T}_r - \mathrm{T}_A)}{\sqrt{\lambda_n}}(\cos(\sqrt{\lambda_n}) - 1) + \frac{h(T_r - T_A)}{\lambda_n(1+hL)}\left(\sqrt{\lambda_n}L\cos(\sqrt{\lambda_n}L) - \sin(\sqrt{\lambda_n}L)\right)}{\frac{L}{2} - \frac{1}{4\sqrt{\lambda_n}}\sin(2\sqrt{\lambda_n}L)}\text{ , } n=1,2,3,.. \tag{S.7}$$

And λ_n is the solution to

$$-\tan(L\sqrt{\lambda_n}) = \frac{\sqrt{\lambda_n}}{h}\text{ , } n=1,2,3,.. \tag{S.8}$$

The solution of the above equation can only be obtained numerically. We solve for the λ_n's using Matlab. Hence, the heat flux q at the boundary x=0 is expressed as,

$$q = k\left(\frac{h(T_A - T_r)}{1+hL} - \sum_{n=1}^{\infty}\sqrt{\lambda_n}B_n e^{-(\lambda_n \alpha_h t)}\right) \tag{S.9}$$

In order to approximate the heat flux q, we shall identify the terms to be considered in equation (S.9). Let $C_n = \sqrt{\lambda_n}B_n e^{-(\lambda_n \alpha_h t)}$ thus the ratio of each subsequent term to the first term is expressed as

$$\frac{C_n}{C_1} = \frac{\sqrt{\lambda_n}B_n}{\sqrt{\lambda_1}B_1} e^{((-\lambda_n+\lambda_1)\alpha_h t)} \tag{S.10}$$

Fig. S1 provides the $\frac{C_n}{C_1}$ plot with respect to time for various n. It can be observed that as n increases, the ratio drops rapidly with increasing time duration. Without loss of generality, we consider the first 100 terms in equation (S.9) to approximate the heat flux as

$$\tilde{q} = k\left(\frac{h(T_A - T_r)}{1+hL} - \sum_{n=1}^{100}\sqrt{\lambda_n}B_n e^{-(\lambda_n \alpha_h t)}\right). \tag{S.11}$$

The total amount of heat that is input to the tissue is

$$\tilde{Q} = \int_0^t \tilde{q}\,(\tau)d\tau = \frac{kh(T_A - T_r)}{1+hL}t + \sum_{n=1}^{100}\frac{kB_n}{\alpha_h\sqrt{\lambda_n}}\left(e^{(-\lambda_n \alpha_h t)} - 1\right) \tag{S.12}$$

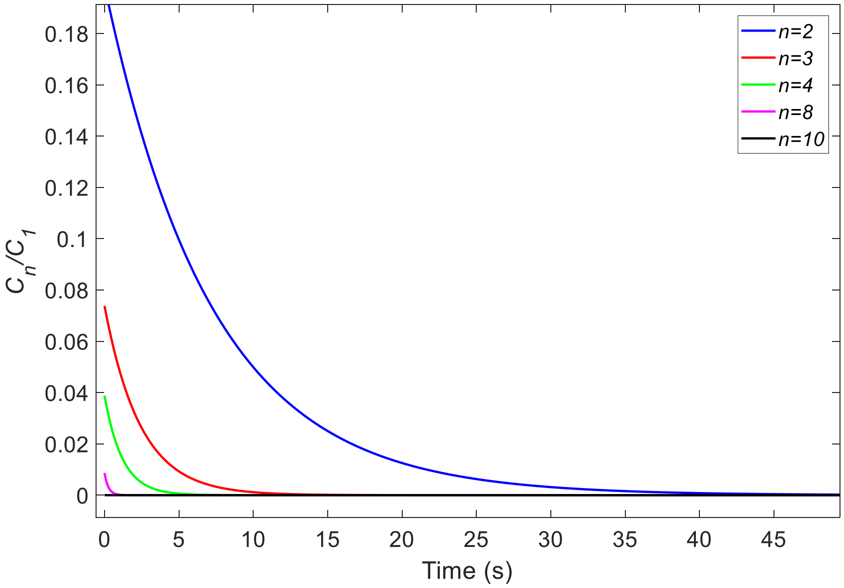

Supplementary Fig. S1: Plots of C_n/ C_1 for n=2,3,4,8, and 100.

Table S1 reports the values of parameters used to estimate the $\tilde{q}$ and $\tilde{Q}$, which are obtained from the literature. T_r =22 ℃ is chosen to be the room temperature. Table S2 lists the values of $\tilde{q}$ and $\tilde{Q}$ for each burn condition used in this study. From Table S2 it can be seen that the magnitude of the flux is mainly affected by the applied temperature for a given duration. Fig. S2. shows the amount of heat uptake for all the burn conditions. It can be seen that the amount of heat increases monotonically. Though the 450 ℉10s condition produces more heat than the 200 ℉30s, the difference in heat generation between these two groups (~235 kJ) is less than those between other burn groups (>580kJ). The similar amount of heat exposure may lead to similar level of burn damage to the tissue, which may explain some similar changes in spectra from these two groups (*e.g.* Fig. 2(b), Fig. 3(c) and (e)).

Supplementary Table S1: Values of parameters used for the estimation.

Parameter	Value
α_h (m^2/s)	2.8×10^{-8} [1]
L (m)	2×10^{-3} [2]
k (W/(m·k))	0.3 [3]
h(W/(m^2·k))	4.2 [4]

Supplementary Table S2: Estimated values of heat flux and the amount of heat for all the burn conditions.

**Burn Temperature**	**Burn Time**	$\tilde{q}$ **(kW/m^2)**	$\tilde{Q}$ **(kJ)**
200 ℉	10s	35.57	420.01
	30s	24.00	1002.39
450 ℉	10s	104.80	1237.79
	30s	70.73	2954.10

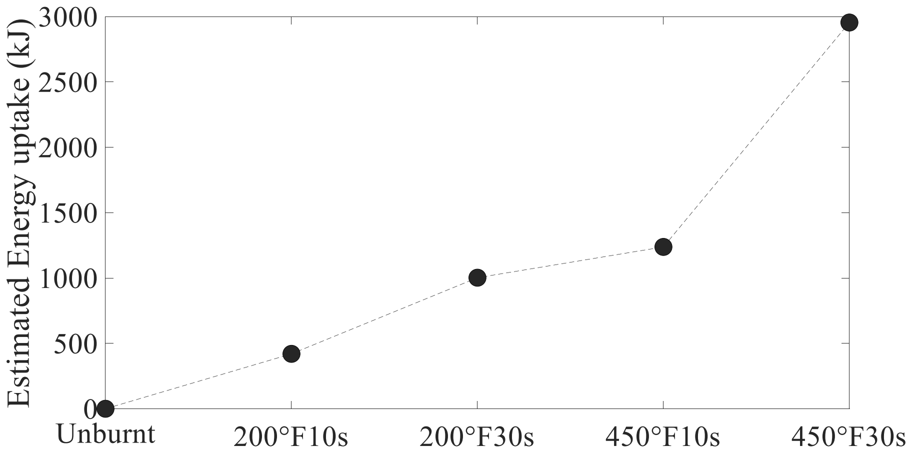

Supplementary Fig. S2: Plots of estimated energy uptake for all the burn groups.

References:

1. Werner, U., Giese, K., Sennhenn, B., Plamann, K. & Kolmel, K. Measurement of the thermal diffusivity of human epidermis by studying thermal wave propagation. Phys. Med. Biol 37, 21–35 (1992).
2. Hendriks, F. Mechanical behaviour of human skin in vivo. Bio-medical Engineering 4, (Nat.Lab., Amsterdam, Netherlands, Unclassified Rep. 2001/820, 1969).
3. Lefevre, J. Studies on the thermal conductivity of skin in-vivo and the variations induced by changes in the surrounding temperature. J. Phys. Théor. 10, 380–388 (1901).
4. Kurazumi, Y. et al. Radiative and convective heat transfer coefficients of the human body in natural convection. Build. Environ. 43, 2142–2153 (2008).
